# Isolated hyperthermic perfusions for cutaneous melanoma in-transit metastasis of the limb and uveal melanoma metastasis to the liver

**DOI:** 10.1007/s10585-023-10234-6

**Published:** 2023-10-16

**Authors:** Anne Huibers, Danielle K. DePalo, Matthew C. Perez, Jonathan S. Zager, Roger Olofsson Bagge

**Affiliations:** 1 Department of Surgery, Sahlgrenska University Hospital, 413 45 Gothenburg, Sweden; 2 Institute of Clinical Sciences, Sahlgrenska Academy, University of Gothenburg, 413 90 Gothenburg, Sweden; 3 Department of Cutaneous Oncology, Moffitt Cancer Center, Tampa, FL USA; 4 Department of Oncologic Sciences, University of South Florida Morsani, College of Medicine, Tampa, FL USA

**Keywords:** In transit metastasis, Uveal melanoma, Isolated limb perfusion, Isolated limb infusion, Isolated hepatic perfusion, Percutaneous hepatic perfusion

## Abstract

Patients with cutaneous melanoma can develop in-transit metastases (ITM), most often localized to limbs. For patients with uveal melanoma that develop metastatic disease, the overall majority develop isolated liver metastases. For these types of metastases, regional cancer therapies have evolved as effective treatments. Isolated limb perfusion (ILP), isolated limb infusion (ILI), isolated hepatic perfusion (IHP) and percutaneous hepatic perfusion (PHP) achieve a high local concentration of chemotherapy with minimal systemic exposure. This review discusses the mechanism and available literature on locoregional treatment modalities in the era of modern immunotherapy.

## Introduction

The incidence of melanoma is rapidly increasing worldwide, and in the Western population one out of every 50 individuals will develop melanoma [1]. Melanoma is a cancer caused by malignant transformation of melanocytes, cells derived from the neural crest cells that migrate to several different sites in the body [2]. Therefore, melanoma may involve various parts of the body, such as the skin (cutaneous melanoma), mucosa (mucosal melanoma), and the eye (uveal melanoma). While most melanomas are detected at an early stage, a proportion of patients will have metastatic disease at the time of diagnosis or develop metastasis at a later stage. The introduction of effective systemic therapies with targeted therapies and immunotherapy has revolutionized clinical management of patients with advanced melanoma, but these treatments can also cause serious side effects [3]. There are particular types of metastases that can develop. Patients with cutaneous melanoma can develop in-transit metastases (ITM), most often localized to limbs [4, 5]. In patients with uveal melanoma that develop metastatic disease, approximately 90% develop isolated liver metastases [6]. For these types of metastases, regional cancer therapies have evolved as effective treatments. Isolated limb perfusion (ILP), isolated limb infusion (ILI), isolated hepatic perfusion (IHP), and percutaneous hepatic perfusion (PHP) achieve a high local concentration of chemotherapy with minimal systemic exposure [7–9]. In this review we give an overview of ILP/ILI for the treatment of ITM in patients with cutaneous melanoma and IHP/PHP for the treatment of liver metastasis in patients with uveal melanoma.

## In-transit metastases

Approximately 5–10% of patients with high risk early-stage melanoma will develop ITM [4, 5]. ITM appear as tumor nodules in the subcutaneous or cutaneous tissues between the primary site and the nearest draining node basin. The metastasis can be of variable size and may or may not be pigmented [10]. The hypothesis is that ITM originates from tumor cell emboli entrapped in dermal lymphatic vessels between primary tumor location and regional lymph nodes [11]. However, ITM is a heterogenous disease and the exact mechanism behind its development is not completely understood. In a study by Jakub et al. clinical predictive factors for the development of ITM include age, lower limb localization, Breslow thickness, ulceration, mitotic rate and positive sentinel node [12]. In the same study, an analysis of 108 genes adjusted for age, Breslow, mitotic rate and localization identified five up-regulated and five down-regulated genes predictive of ITM recurrence, where CXCL1/8-CXCR1/2 pathways seemed most important, and integrin/growth factor receptor signaling was less important [12]. Interestingly, some patients with ITM progress rapidly with distant metastases, while others stay stable with regional disease for years. This heterogeneity makes an individual treatment approach necessary, aiming for both locoregional control and reducing the risk for distant recurrence. Surgical resection is usually recommended for patients with limited and resectable disease. For patients in which surgical treatment is not deemed feasible, regional therapies (including intralesional therapies, ILP/ILI, radiotherapy or topical therapy) or systemic therapies can be considered [7–9, 13].

### Systemic treatments

Systemic treatment options for unresectable stage III disease are identical to those available to patients with stage IV melanoma. Targeted therapy with BRAF and MEK inhibitors, or immune checkpoint blockade with a PD-1 antibody (nivolumab or pembrolizumab) as monotherapy, or PD-1 and CTLA-4 antibodies (nivolumab and ipilimumab) in combination, have been shown to improve progression-free survival (PFS) and overall survival (OS) for patients with advanced melanoma [14–18]. Interestingly, in the immunotherapy registration trials patients with unresectable stage III disease were included, however in a study analyzing these trials there were no patients with ITM identified, so no prospective randomized data on the efficacy of immunotherapy for ITM exists [19]. There have been suggestions that systemic therapies are less effective than locoregional therapies in patients with ITM, on the other hand, they can have the potential benefit that undetected distant micro-metastatic disease is being treated simultaneously. A few analyses have reported on the response rates of systemic therapies in ITM, and within these studies, a total of 474 patients were treated with anti-PD1, anti-CTLA4 or both in combination [20–23]. Overall response rates (ORR) varied from 31 to 62%, with complete response (CR) rates between 13 and 62%. There is even less data on response rates for patients treated with targeted therapy, Zaremba et al. reported on 19 patients with ITM treated with either BRAF inhibition (n = 9) or BRAF/MEK inhibition (n = 10). Results showed an ORR of 63% and CR rate of 26% [23]. Current clinical guidelines, such as those published by the American Society of Clinical Oncology (ASCO) and European Society for Medical Oncology (ESMO), suggest systemic therapy as well as regional therapies as treatment options for patients with ITM [24, 25].

### Isolated limb perfusion

**Fig. 1:**
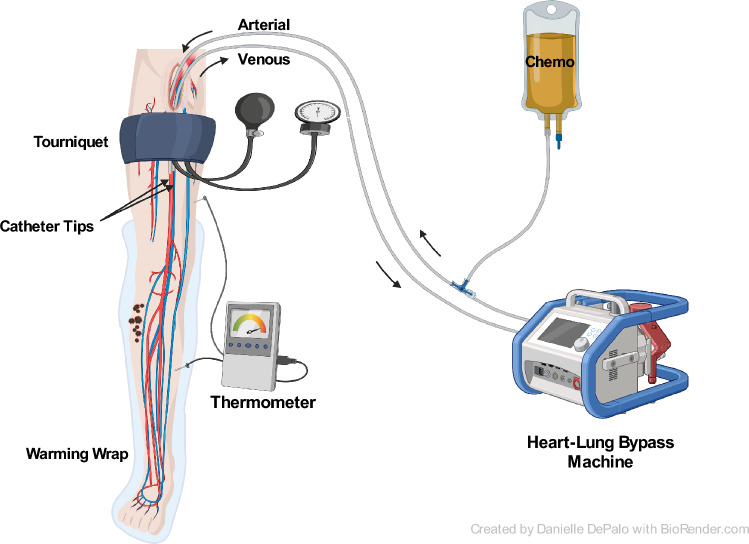
Isolated limb perfusion (ILP)

The technique of ILP was first described by Creech and Krementz in 1958 [26]. The treatment concept consists of surgical isolation of the extremity and the connection of the circulation to an extracorporeal heart–lung machine [Fig. 1]. This allows regional administration of heated chemotherapy at concentrations that would not be possible systemically [7]. To further minimize potential systemic side effects of the chemotherapy, continuous leakage monitoring and adjustment of flow rate are performed throughout the procedure. The most common chemotherapy agent for perfusion is melphalan, but when bulky disease is present, or in patients undergoing a repeat procedure, the addition of tumor necrosis factor-alpha (TNF-alpha) can be of additional value. However, TNF-alpha is available mainly in Europe and not in e.g., North America or Australia. The procedure has proved to be safe and effective, with a CR rate of approximately 60% and an ORR of 90% [27]. ILP can also safely be repeated, achieving similar high response rates and comparable toxicity as for first-time ILP procedures. Repeat ILP procedures is mainly indicated for patients who already showed a CR after the first ILP, in patients not responding other treatment options should be considered [PMID 30617871].

One study has examined the effect of BRAF-mutational status, but the response to ILP was similar independent of BRAF status [28]. The treatment is also suitable for elderly patients, and ILP can also be repeated safely for those with recurrent disease [28–30]. However, there exist no studies showing that ILP reduces the risk of distant metastasis, suggesting that ILP primarily should be seen as a locoregional treatment [31, 32]. Two recent papers assessed whether outcomes after ILP were influenced by previous immunotherapy [33, 34]. Davies et al., published a retrospectively analysis of 97 patients that had undergone ILP, out of which 16 had received prior immunotherapy. In the study of Holmberg et al., 218 patients were included, of which 20 had received and failed prior immunotherapy. In the later study, there was no reduced effect of ILP when comparing the two cohorts (50% vs 46%). Whereas Davies et al. showed a significant difference in complete response rate after ILP, in favor of those that were immunotherapy-naïve (6% vs 47%, p = 0.0018). There was no significant difference between age, sex distribution and positive lymph node disease at presentation between the groups. A possible explanation for these contrasting findings may be a difference in referral policies between institutions.

Currently, we lack international treatment algorithms on how to treat extensive in transit metastasis. Surgical resection, when feasible, continues to represent a standard approach for patients with localized low disease burden. Patients with more extensive disease benefit from ILP with high response rates, and ILP is probably also a valid treatment option with high response rates in patients that have previously failed systemic immunotherapy.

### Isolated limb infusion

**Fig. 2:**
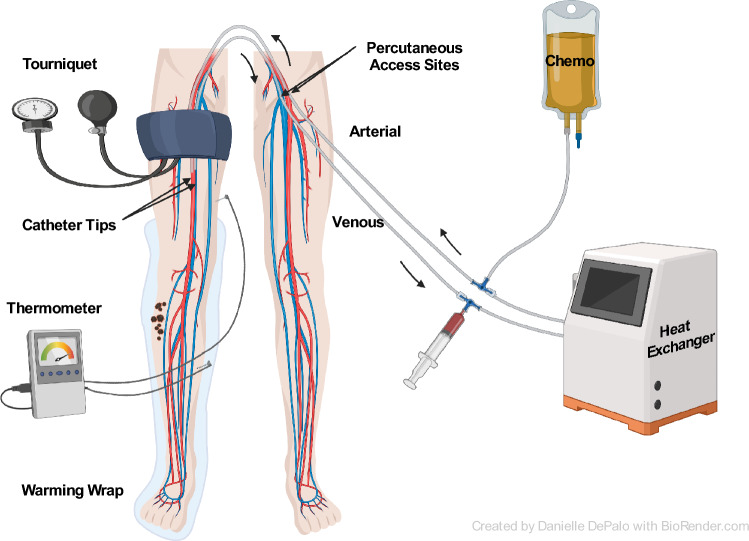
Isolated limb infusion (ILI)

In 1996, Thompson et al. described the novel technique of isolated limb infusion (ILI), a technically simpler alternative to ILP. Vascular access is gained by placing arterial and venous catheters by interventional radiology via the contralateral groin [Fig. 2]. The correct placement of the catheters is confirmed by fluoroscopy. The patient is systemically heparinized prior to tourniquet occlusion. Melphalan and dactinomycin are typically used, which are dosed according to limb volume corrected for ideal body weight to limit toxicity. The chemotherapy is infused manually via a syringe during 20–30 min [35–38]. A large multi-center report of long-term efficacy of ILI showed an impressive ORR of 64% and a CR of 29%, with the complete responders after ILI having more than 6 years median OS after ILI [39]. Other single and multi-institutional studies show efficacy and safety also in elderly, showing an ORR of nearly 70% after ILI in patients older than 80 years [40] (see Table 1).

**Table 1:** Comparison of technique and outcome of ILP and ILI [41–45]

	Isolated limb perfusion (ILP)	Isolated limb infusion (ILI)
Technique	Open surgical exposure of vessels	Percutaneous catheterization
	Duration approximately 3 h	Duration approximately 1.5 h
	Perfusion approx. 60 min	Infusion approx. 20–30 min
	Heart–lung machine needed	No need for heart–lung machine
	No fluoroscopy needed	Fluoroscopy needed
	General anesthesia	Possible with regional anesthesia
	TNF-alpha	No TNF-alpha
	Leakage monitoring recommended	No leakage monitoring
	Repeatable	Repeatable
Outcomes		
Wieberdink grade IV toxicity	3–4%	0–1%
Overall response rate	80–81%	43–53%
Complete response rate	55–57%	24–50%
Median overall survival	33–40 months	32–46 months

No randomized comparisons between ILI and ILP have been reported, but there appear to be differences in both outcome and complication rates, whether these differences are significant is yet to be assessed. Overall response rates for ILP and ILI have ranged from 40 to 90%, a higher ORR is reported for ILP in most of the literature [31, 41–43]. In a large, retrospective review of 225 patients undergoing ILP or ILI, ILP had a higher ORR (81 vs. 43%, *p* < 0.001) with a similar regional toxicity between the treatments, with the exception that ILP was more likely to be associated with the rare complication of limb loss (3 vs. 0%) [41]. A limitation of this comparison is that it included a high selection bias between the procedures, not stratifying for burden of disease which is known to correlate with outcomes after regional treatments [44]. In a study comparing two large-volume, single-institution series of ILP and ILI including 203 patients with ITM, adjusting for known predictive factors, ILP offered higher overall (80% vs. 53%) and complete response rates (60% vs. 29%), but this did not translate into any prolonged overall survival [45]. Importantly, in this more modern series there was no major difference in toxicity, and no patient developed the complication of limb loss.

Taken together, ILP seems to have higher response rates but also potentially higher toxicity. Few centers offer both ILP and ILI, and taking into consideration that both techniques require special knowledge and a multi-disciplinary team, the treatment to recommend is likely the treatment that the center is used to perform. Both procedures can be repeated multiple times with similar response rates, where ILP might be more technically challenging but where some institutions have the possibility to also add TNF-alpha, potentially increasing the response rates even further.

### Combinatorial treatments

During ILP, the isolated limb is only treated with melphalan for 60 min; however, in patients with a complete or partial response, it usually takes several months before metastases disappear completely, implying that the cytotoxic effect is not solely responsible for the observed tumor regression. A potential explanation would be that the response is at least partially immune-mediated. Although there is limited translational data, recent studies support the potential link between ILP-induced cellular immunity and the clinical benefit of ILP. ILP triggers an increase of activated T cells in peripheral blood, and CR rates following ILP are associated with the presence of activated CD8 + T cells prior to ILP [46–48]. In a murine model of melanoma, the combination of chemotherapy and CTLA-4 blockade induced a cellular shift in the local tumor microenvironment, with infiltrating CD8 + and CD4 + T cells increasing the CD8 + /Foxp3 T-cell ratio and leading to an improved survival [49]. In a phase II trial including 26 patients with advanced melanoma treated by ILI with melphalan and the CTLA-4 inhibitor ipilimumab, the ORR was 85%, including 62% CR [49]There is also preclinical evidence that PD-1 inhibition could potentially increase the efficacy of ILP, and these findings further support the rationale behind ongoing studies, evaluating the combination of regional therapy and immunotherapy [50]. Two examples of ongoing studies are the Nivo-ILP trial (ClinicalTrials.gov: CT03685890) and NIVEC (ClinicalTrials.gov: NCT04330430), which are recruiting patients with in-transit metastasis, examining the combination of ILP and nivolumab, and T-VEC and nivolumab, respectively. Another phase I/II trial, the TITAN trial, is evaluating the safety of using T-VEC in combination chemotherapy administered via ILP (ClinicalTrials.gov: NCT03555032).

## Uveal melanoma

### Clinical background

Uveal melanoma derives from melanocytes located in the choroid (90%), ciliary body (6%) and iris (4%), which are all structures of the uveal tract [51]. In terms of pathogenesis, incidence, clinical presentation, and response to treatment, the behavior is very different compared to cutaneous melanoma. While ultraviolet (UV) light radiation is an established risk factor for the development of cutaneous melanoma, wavelengths of the UV radiation cannot penetrate and reach the posterior eye where the choroid is located [52]. Ultraviolet light is therefore not implicated in the pathogenesis, with the exception of iris melanomas, which arise from a region of the eye exposed to UV light [53]. The incidence trend of uveal melanoma has different from cutaneous melanoma, where the incidence over the past decades has remained stable, ranging from 4.9 to 10.9 per million [54, 55]. At the time of diagnosis, the majority of patients present with symptoms (87%) ranging from various visual disturbances to visual loss, whereas asymptomatic patients are usually diagnosed during a routine eye examination [56]. Historically, the treatment of uveal melanoma has usually consisted of enucleation, which is still an appropriate treatment in the presence of a large tumor. However, there has been a shift towards eye-preserving modalities, based on the results of the Collaborative Ocular Melanoma Study (COMS) where no survival benefit was showed in patients treated with enucleation compared to brachytherapy [57]. This technique is now accepted as treatment for small and medium sized tumors, with a 5-year local recurrence rate of less than 5% [58].

### Distant metastasis

Despite the relatively good response of the primary lesion, patients with uveal melanoma have a poor prognosis; distant metastasis develop in 25–31% within 5 years, 34–45% within 15 years, and 49% within 25 years of diagnosis [6, 59]. The liver is the most common site of metastasis (93%), but some patients could have additional metastases including the lungs (24%), bones (16%) and soft tissues (11%) [60]. Once the disease has metastasized, the median OS is less than one year, with only 8% of patients surviving at two years [61]. Several clinical, histological, and molecular factors are associated with the risk of developing stage IV disease. The American Joint Committee on Cancer classification system for uveal melanoma is based on clinical features, in which a larger tumor size, ciliary body involvement, and extraocular extension are associated with a higher risk of metastasis. Besides clinical features, advances in the understanding of molecular mechanisms underlying uveal melanoma have enhanced prognostication. Currently, the most clinically significant genetic alterations are mutations in the genes encoding the BRCA-1 associated protein 1 (BAP-1). These mutations are found in 47% of primary uveal melanoma and 84% of metastatic patients, and there is an association between BAP1 mutation and a poor prognosis [62].

The reason why uveal melanoma metastasize primarily to the liver is far from understood. In contrast to conjunctival melanoma, uveal melanomas are known to metastasize hematogenous, and a common explanation is the lack of lymphatics within the eye. Regional lymphatic metastases are exceptionally rare and are associated with extraocular extension or orbital recurrences where the tumors have the possibility to invade into the lymphatic system. However, new findings have questioned the lack of lymphatic drainage from the eye, whether this affects the metastatic route is still unknown [63]. An early observation by Zimmerman et al. in patients with uveal melanoma was that there was a peak in mortality 2 to 3 years after primary diagnosis and enucleation, and one hypothesis was that the surgery itself released tumor cells causing this effect. This was one of the reasons why the COMS-trial of enucleation compared to brachytherapy was initiated [64]. An alternative hypothesis was that uveal melanoma cause early micro-metastatic disease, and by calculating the doubling times of both the primary tumor and the metastases, Eskelin et al. created a mathematical model showing that if a primary tumor would cause metastases, that would have been initiated approximately 3 years before diagnosis, and it would take an additional 2 years for the liver metastases to become clinically detectable [65]. Indeed, micro-metastatic disease have been identified in patients, and two different growth patterns have been proposed. In the first pattern the sinusoidal space is included (infiltrative growth pattern), and in the second the growth is located in the periportal area (nodular growth pattern) [66]. Murine models have shown that modification of immunogenic factors, with e.g., the activation of natural killer (NK) cells by interferon alpha 2b, decreases micro-metastasis in the liver [67]. Also, non-immunogenic factors are of importance, e.g., where pigment epithelium-derived factor (PEDF) have been shown to prevent progression of liver metastasis [68]. A novel and interesting concept is the role of primary tumor derived extracellular vesicles (EVs) and their ability to create pre-metastatic niches within the liver. E.g., EVs from colorectal cancer induce transforming growth factor beta (TGF-β) mediated epithelial to mesenchymal transition of hepatocytes [69]. Proteomic analysis of EVs from uveal melanoma cell lines have identified several proteins associated with organotropic metastasis to the liver, including TGF-β [70]. Another study isolated EVs from different uveal melanoma cell lines, these EVs were then used to treat fibroblasts and the results showed increased proliferation, migration, invasion and a general acquisition of malignant characteristics [71].

### Systemic treatments

Although multiple systemic treatments have shown efficacy in cutaneous melanoma, the results in patients with uveal melanoma are disappointing. While most evidence comes from single arm studies, some randomized trials have been performed [72–78]. For chemotherapy, most studies have used dacarbazine, temozolamide, or fotemustine, all with limited efficacy as single-agent treatments, with ORRs ranging between 0 and 10% [73, 75, 76]. Evidence from preclinical studies suggests that targeting MEK has efficacy against uveal melanoma cells in vitro, which has led to three randomized controlled trials evaluating the MEK inhibitor selumetinib, either alone or in combination with chemotherapy [73, 74, 76, 79]. Although two of the trials showed a significant improvement in PFS, there was no improvement in OS [73, 76].

The tumor mutational burden in uveal melanoma is lower compared to cutaneous melanoma, which may explain its low sensitivity to checkpoint inhibition [80]. Efforts have been made to enhance the efficacy of single-agent immunotherapeutics, for example, by adding epigenetic therapy using the histone deacetylase (HDAC) inhibitor entinostat to upregulate the expression of immune signaling components in melanoma cells. In the PEMDAC trial, this agent was combined with pembrolizumab and showed a median PFS of 2.1 months and a median OS of 13.4 months [81]. Two single-armed trials examined the combination of CTLA-4 and PD-1 inhibition (ipilimumab and nivolumab) with a median OS of 12.7 and 19.1 months, respectively [82, 83].

The first treatment to show a prolonged OS in a phase III randomized trial was tebentafusp, a bispecific antibody consisting of an affinity-enhanced T-cell receptor fused to an anti-CD3 effector, which redirects T cells to target glycoprotein 100–positive cells. The trial randomized patients to receive either tebentafusp or investigator’s choice treatment with single-agent pembrolizumab, ipilimumab, or dacarbazine [72]. In this trial, including a total of 378 patients, treatment with tebentafusp resulted in significantly longer OS (21.7 vs 16.0 months). Interestingly, the benefit in PFS or tumor response was low with a median PFS 3.3 months in the tebentafusp group vs. 2.9 months in the control group [72]. This implies a clinically important effect on outcome for patients, even without a radiographically significant decrease in tumor size.

### Loco-regional treatments

Using the same rationale as for isolated limb perfusion, isolated hepatic perfusion (IHP) is a treatment modality that exposes liver metastases to a high local concentration of melphalan with minimal systemic toxicity. IHP was originally designed as an experimental model in canines and later Ausman et al. developed the technique in a porcine model [26, 84]. In 1960, the outcome of the first five patients treated with IHP using nitrogen mustard for 60 min was reported [85]. Since then, IHP has been clinically evaluated in several studies, mainly for liver metastases derived from colorectal cancer, melanoma, and neuroendocrine tumors, but also for primary hepatic malignancies [86]. IHP is a major and complex surgical intervention. Since the first report by Ausman et al., there have been many developments in surgical technique that have decreased morbidity and improved response rates. One of the differences between studies has been whether to shunt the portal vein, include it in the perfusion, or to clamp it.

The only randomized controlled phase III trial investigating IHP, the SCANDIUM trial, included 93 patients with previously untreated isolated liver metastases from uveal melanoma. The patients were randomized to receive a one-time treatment with IHP with melphalan or best alternative care (control group). In the control group, 49% of the patients received chemotherapy, 39% immune checkpoint inhibitors, and 9% other locoregional treatments. In an intention-to-treat analysis, the ORR was 40% compared to 4.5% favoring IHP, and this also translated to a benefit in median PFS (7.4 vs. 3.3 months) [87].

In the early 1990s, three independent groups developed a novel percutaneous hepatic perfusion (PHP) system using extracorporeal chemofiltration. The technique combined a conventional hepatic artery infusion with a dual-balloon vena cava catheter collecting the outflow from the liver. The venous outflow was then connected to an extracorporeal venous bypass circuit, including a carbon filter, to recover any of the drug that was not absorbed by the liver [88, 89]. A phase I dose escalation study using melphalan including 28 patients, showed an ORR of 30%, and in ten patients with melanoma, the response rate was 50% [90]. This finding lead to the initiation of a phase III study, randomizing 93 patients to either PHP or best alternative care (BAC). The median PFS was 5.4 months for PHP compared to 1.6 months in the control group; however, there was no difference in median OS (10.6 months vs. 10.0 months), potentially due to a high rate of crossover from the control group to the PHP group [91].

A recent phase III study, the FOCUS trial, compared PHP to the investigator’s choice of transarterial chemoembolization, ipilimumab, pembrolizumab, or dacarbazine in patients with hepatic-dominant disease. The trial started as a randomized trial where 43 patients were randomized to PHP and 42 to the control arm, but due to enrollment concerns the control arm was stopped and another 59 patients was assigned to the PHP arm. The ORR was 35% in patients receiving PHP compared 12.5% in the control, which translated to significantly prolonged PFS (9.0 vs. 3.1 months) [92]. Both the SCANDIUM and the FOCUS trial show similar response rates and prolongation in PFS, further supported by data from a recent meta-analysis, where the hepatic PFS was 10.0 vs. 9.5 months and the OS 17.1 vs. 17.3 months when comparing IHP and PHP for patients with uveal melanoma liver metastases. However, there was a higher complication rate (39.1% vs. 23.8%) and a higher 30-day mortality (5.5% vs. 1.8%) for patients treated with IHP compared to PHP [9]. An interesting development is the combination of IHP or PHP with systemic immunotherapy, and ongoing studies are currently investigating the combination of CTLA-4 and PD-1 inhibitors with either PHP (CHOPIN trial ClinicalTrials.gov NCT04283890) or IHP (SCANDIUM-II trial ClinicalTrials.gov NCT04463368).

## Biomarkers for response

For patients with ITM undergoing ILP or ILI, there is yet no established molecular biomarkers that predicts response. An interesting research field is immunological factors associated with response, and early data have suggested that patients achieving a CR after ILP had higher counts of CD3^+^CD8^+^CD45RA^+^ T cells as well as activated CD3^+^HLA-DR^+^ T cells before treatment [48]. For patients with uveal melanoma liver metastases undergoing treatment with IHP/PHP, there are similarly no established molecular biomarkers. Data has shown a correlation between OS and a high infiltration of CD8 + T cells in metastases, and an activated immune cell profile in peripheral blood, in patients treated with IHP [47]. Research is ongoing using RNA and DNA sequencing trying to identify patterns of response and progression after IHP [78].

## Conclusion

For patients with cutaneous melanoma ITM or liver metastasis from uveal melanoma, isolated hyperthermic perfusion is an effective treatment modality with high response rates. There is also emerging evidence that combinatorial treatments with modern immunotherapy might enhance efficacy further. For patients with melanoma in-transit metastases, where only approximately 30% achieve a complete response after modern immunotherapy, there is definitely a future for regional treatments, either by intralesional therapies or ILP/ILI. For patients with uveal melanoma liver metastases, IHP/PHP achieves high response rates with marked PFS benefits, but in order to substantially improve overall survival the addition of systemic treatments will be of the highest importance.
